# Prediction of pre-eclampsia and eclampsia complications using PIERS

**DOI:** 10.6026/9732063002001383

**Published:** 2024-10-31

**Authors:** Paliwal Paliwal, Nitin S. Kshirsagar, Rajkumar P. Patange

**Affiliations:** 1 Department of Obstetrics and Gynecology, Krishna Institute of Medical Sciences, Karad - 415110, Maharashtra, India

**Keywords:** Preeclampsia, eclampsia, preeclampsia integrated estimate of risk score, adverse maternal outcome, feto-maternal outcomes

## Abstract

Preeclampsia (P-EP) and eclampsia (EP) result in 50,000 maternal deaths annually. Therefore, it is of interest to assess P-EP and EP complications using P-EP integrated estimate of risk score (PIERS). We categorized 60 patients into two groups: group 1, which included 14 patients with Adverse Maternal outcome (AMO) and group 2, which included 46 patients without AMO. They were evaluated using different laboratory investigations and PIERS for adverse feto-maternal outcomes (AFMO). There is significant influence of maternal health conditions on fetal outcomes, with a considerable percentage of new-borns needing intensive care and displaying indications of distress immediately after delivery. Thus, results support the incorporation of the PIERS score into standard obstetric care for patients with P-EP and associated conditions.

## Background:

Preeclampsia (P-EP) is a multifaceted condition that arises from placental dysfunction and its clinical manifestations are the consequence of several endothelial abnormalities caused by an imbalance of angiogenic and anti-angiogenic substances [1]. The diagnostic criteria consist of a systolic blood pressure of 140 mmHg or above and/or a diastolic blood pressure of 90 mmHg or greater. These criteria are associated with significant proteinuria or clinical or laboratory indications of maternal organ failure [1]. The last resort is to end the pregnancy (and deliver the placenta); however, expectant care, particularly for early-onset PE, may be an option to enhance perinatal outcomes with close monitoring, provided that the mother's and the fetus's survival permits such follow-up [2]. P-EP significantly increases maternal and fetal morbidity and mortality, affecting up to 5% of births worldwide [3, 4]. P-EP may result in maternal complications like abruptio placentae and severe renal failure, as well as fetal complications including small-for-gestational-age newborns, respiratory distress and stillbirth, among others [5]. There are two types of P-EP (*i e.*,) Early-onset and late-onset. A precise reason of P-EP is still unclear; however research has shown that the development of its two types may have distinct origins [6, 7]. Some researchers propose that late-onset P-EP is caused by a maternal tendency to develop arterial disease, leading to excessive inflammation during pregnancy (maternal P-EP). On the other hand, early-onset P-EP is believed to be caused by a problem with the invasion of tropho-blast cells into the mother's spiral arteries, resulting in inadequate remodeling of the blood vessels (placental P-EP) [6]. Late-onset P-EP is associated with more catastrophic effects, such as impaired foetal development, despite the fact that it becomes more prevalent later in pregnancy [5]. Early-onset P-EP is difficult to manage since presently, the only treatment for P-EP is delivery, which might result in preterm birth, which has major effects. This makes the management of preeclampsia a challenging challenge [5, 8]. As a result, it is recommended to postpone delivery whenever it is feasible; however, because the mother is also more likely to encounter problems, it is unclear how long expectant management should continue. It would be extremely beneficial to be able to forecast the risk of maternal problems in order to guide the management of women who have been referred to care facilities with early-onset preeclampsia with the intention of guiding their care [3, 9]. The goal of the comprehensive PIERS model is to notify clinicians about the need for further treatment and the timing of the delivery in women who are at risk of unfavorable maternal outcomes [8]. The PIERS is calculated using factors such maternal blood oxygen saturation, platelet count, creatinine and aspartate aminotransferase levels, gestational age at the time of diagnosis and the presence of chest pain and dyspnea [10]. Recent validation of PIERS has shown that it predicts unfavorable maternal outcomes in the near future quite well [10]. Therefore, it is of interest to report PIERS for the prediction of complication about P-EP and EP.

## Materials and Methods:

The current hospital based prospective observational clinical study was conducted over a period of one and a half years in the department of Obstetrics and Gynaecology with 60 patients in total with the help of investigations like CBC, blood sugar level, PC INR & PT, serum bilirubin, SGPT, SGOT, alkaline phosphatase, LDH & A: G ratio, blood urea, serum creatinine, serum electrolytes, uric acid & urine albumin and pulse oximetry. Then, full PIERS calculator was utilized to calculate the risk of adverse feto-maternal outcomes (AFMO). Patients with gestations of less than 34 weeks received 2 doses of betamethasone (12mg each, 24 hours apart). Patients with imminent EP were administered magnesium sulfate (Mg_2_SO_4_) and monitored intensively to prevent maternal and fetal complications. Antihypertensive medications, such as labetalol and nifedipine, were used to manage hypertension, with dosages adjusted according to severity. The mode of pregnancy termination depended on the gestational age, cervical favorability and urgency of termination. Cervical priming agents such as PGE2 gel were used if the cervix was unfavorable. Caesarean sections were performed based on obstetric indications, including fetal distress and failure of induction.

## Interpretation of PIERS:

## Low Risk (<2.5%):

Patients with a PIERS score indicating less than 2.5% risk are considered to be at low risk for severe complications. These patients typically require standard monitoring and management according to established P-EP protocols.

## Intermediate Risk (2.5-30%):

Patients with a PIERS score indicating a 2.5- 30% risk require closer monitoring and may need additional interventions to prevent the progression of the disease. Management strategies might include more frequent follow-ups and targeted therapeutic measures.

## High Risk (>30%):

Patients with a PIERS score indicating more than 30% risk are at high risk for severe complications such as eclampsia, HELLP syndrome, and organ damage. These patients often require intensive monitoring, hospitalization, and aggressive treatment to mitigate the risks to both mother and fetus.

## Clinical Utility of PIERS:

## Risk stratification:

The PIERS score helps clinicians categorize patients based on their risk of adverse outcomes, enabling targeted management strategies.

## Early intervention:

By identifying high-risk patients early, healthcare providers can implement timely interventions, potentially improving maternal and fetal outcome.

## Resource allocation:

The PIERS score assists in the efficient allocation of healthcare resources by identifying patients who need intensive monitoring and treatment.

## Patient counseling:

The score provides a quantitative basis for counseling patients about their risk of complications, aiding in shared decision-making.

## Inclusion criteria:

[1] Those who were diagnosed with P-EP & EP.

[2] Patients with HELLP syndrome.

## Exclusion criteria:

Those who experienced an adverse outcome prior to fulfilling PIERS criteria

## Statistical analysis:

Statistical significance was determined using chi- square test for categorial variables while unpaired t test was used for continuous variables. P-value <0.05 was considered at statistically significant at 95% confidence interval.

## Result:

Table 1 shows that, 25-30 years age group had the highest number of AO, with 7 women (50.0%) affected, compared to 19 women (41.3%) without AO. The p-value for the distribution across these age groups was 0.7244, indicating no statistically significant difference in adverse maternal outcomes (AMO) based on age. Table 2 shows that, p-value was 0.854, indicating no statistically significant difference in AMO between primigravida and multigravida women. This suggests that parity may not be a significant factor in predicting AMO in this study population. Table 3 shows that, right upper quadrant or epigastric pain was reported by 35.7% of women with adverse outcomes, significantly higher than the 10.9% without as the p value was 0.0192. Chest pain or dyspnea was reported by 28.6% of women with adverse outcomes compared to 10.9% without as the p value was 0.0401. Table 4 shows that, among women with AO, 10 (71.4%) had elevated AST levels (>40 U/l), compared to 15 (32.6%) of women without AO, with a significant as shown as p-value was 0.0098. Elevated serum creatinine levels (>1.1 mg/dl) were present in 8 (57.1%) of women with adverse outcomes, significantly higher than the 8 (17.4%) in women without AO as the p value was 0.0032. Dipstick proteinuria (≥1) was observed in 7 (50.0%) of women with AO compared to10 (21.7%) of those without as the p value was 0.0399. Lastly, a platelet count of less than 1.5 lacs was seen in 6 (42.9%) of women with AO, versus 8 (17.4%) in those without AO as the p value was 0.0485. Table 5 shows that, between 34-36 weeks showed more AMO was 42.9% of women with AO were diagnosed, while 28.3% of those without AO were diagnosed in this period as the p value was 0.0456 suggests that GA at the time of presentation is a significant factor in predicting AMO in this study population. Table 6 shows that, there was a noticeable distribution of deliveries and AO across different GA, the differences were not statistically significant as the p value was 0.9337. Table 7 shows that, the MSBP at the time of admission was significantly higher in the AO group (170.2 ± 16.5 mmHg) compared to the no AO group (158.2 ± 17.2 mmHg), as the p value was 0.0246. Similarly, the MDBP was higher in the AO group (104.9 ± 9.3 mmHg) than in the no AO group (98.6 ± 8.9 mmHg), as the p value was 0.0253. The MABP was also significantly elevated in the AO group (127.6 ± 11.7 mmHg) compared to the no AO group (118.1 ± 10.8 mmHg), as the p value was 0.0064.

Table 8 shows that, among the women with AO, 8 (57.1%) required Mg_2_SO_4_, while only 7 (15.2%) of women without AO required it, with a significant difference as the p value was 0.0015. Conversely, 6 (42.9%) of women with AO did not require Mg_2_SO_4_, compared to 39 (84.8%) of women without AO. Table 9 shows that, headaches were significantly more common in women with AO 9 (34.6%) compared to those without 4 (11.8%), as the p value was 0.0332. Chest pain or dyspnea was reported by 7 (26.9%) of women with AO, significantly higher than the 2 (5.9%) of women without AO as the p value was 0.0237. Epigastric pain was noted in 7 (26.9%) of women with adverse outcomes significantly higher, compared to 1 (2.9%) of those without as the p value was 0.0522. Table 10 shows that, a PC of less than 1.5 lakhs was observed in 11 (42.3%) of women with AO, compared to 5 (14.7%) of those without AO as the p value was 0.0165. Elevated AST levels (>40 U/l) were present in 13 (50.0%) of women with AO, compared to 12 (35.3%) of those without as the p value was 0.1088. Elevated serum creatinine levels (>1.1 mg/dl) were found in 12 (46.2%) of women with AO, significantly higher than the 4 (11.8%) in those without as the p value was 0.0028. Dipstick proteinuria (≥1) was observed in 10 (38.5%) of women with AO, compared to 7 (20.6%) of those without as the p value was 0.1281. Table 11 shows that, among participants delivered vaginally spontaneously (VS), 5 (19.2%) experienced APO, whereas 21 (61.8%) did not. For those delivered via induced vaginal delivery, 13 (50.0%) had AO, compared to 10 (29.4%) who did not. In the caesarean section group, 7 (26.9%) had AO, while 4 (11.8%) did not. This difference was statistically significant (P = 0.0079). This suggests a statistically significant association between VS delivery and APO in this dataset.

Table 12 shows that, significant correlations across multiple factors. GA less than 34 weeks was associated with a higher incidence of AO (14.3% vs. 8.7%) as the P value was 0.0456. Presence of chest pain significantly increased the likelihood of AO (28.6% vs. 10.9%) as the P value was 0.0401. Participants with SpO2 levels below 94.9% exhibited a substantially higher rate of AO compared to those with higher SpO2 levels (<0.001). Lower PC (<150,000 cumm) were linked to AO (42.9% vs. 17.4%) as the P value was 0.0485, as were elevated serum creatinine levels (>1.1 mg/dL) (57.1% vs. 17.4%) as the P value was 0.0032 and elevated serum AST (>40 IU/L) (71.4% vs. 32.6%) as the P value was 0.0098. These findings highlight the predictive value of Full-PIERS parameters in identifying maternal health risks, underscoring their potential utility in clinical practice for early intervention and improved management of maternal complications. Table 13 shows that, among the 60 women included, 53 had a PIERS score of less than 30, with 9 (16.98%) experiencing AO. In contrast, 7 women had a PIERS score of 30 or higher, among whom 5 (71, 42%) encountered AO. Overall, the percentage of women experiencing AO was 23.3%. Table 14 the sensitivity of the PIERS score was found to be 35.71% (12.76% to 64.86%). Specificity was higher at 95.65% (85.16% to 99.47%). The positive likelihood ratio was calculated as 8.21 (95% CI 1.78 to 37.81), indicating that a positive PIERS score is associated with a 8.21 times higher likelihood of an AO. Conversely, the negative likelihood ratio was 0.67 (95% CI 0.45 to 1), suggesting that a negative PIERS score reduces the likelihood of an AO by 0.67 times. These validity statistics underscore the utility of the PIERS score as a diagnostic tool for identifying women at risk of AMO, with a particular strength in ruling in the presence of risk.

Table 15 shows that, AO include thrombocytopenia, eclampsia, postpartum hemorrhage (PPH), abruptio placentae, HELLP syndrome, intensive care unit (ICU) admission, ionotropic support, acute renal failure, requirement of blood or blood products, pulmonary edema, cerebral vascular accident (CVA) and mortality. Thrombocytopenia was the most prevalent adverse outcome, affecting 6 cases (42.9%), followed by eclampsia and PPH, each affecting 5 cases (35.7%). Abruption of placenta and HELLP syndrome affected 4 cases each (28.6%), while ICU admission, ionotropic support and acute renal failure were observed in 4 cases each (28.6%) Other outcomes included the need for blood or blood products in 2 cases (14.3%), pulmonary edema in 1 case (7.1%) and a cerebral vascular accident (CVA) in 1 case (7.1%). Notably, there were no reported maternal deaths in the study. Table 16 shows that, O include NICU (Neonatal Intensive Care Unit) admission, meconium-stained liquor (MSL), APGAR score less than 4 at 5 minutes, fetal growth restriction, prematurity, stillbirth and neonatal death. NICU admission was the most common adverse fetal outcome, affecting 14 cases (53.8%), followed by meconium- stained liquor (MSL) in 11 cases (42.3%). 10 cases (38.5%) experienced an APGAR score less than 4 at 5 minutes, indicative of compromised neonatal health immediately after birth. Fetal growth restriction was noted in 9 cases (34.6%), while 7 cases (26.9%) involved premature birth. There was one reported case each of stillbirth (3.8%) and neonatal death (3.8%). These findings underscore the significant impact of maternal health conditions on FO, with a substantial proportion of infants requiring intensive care and exhibiting signs of distress shortly after birth.

## Discussion:

The model, which was developed using a prospective cohort of 2023 women hospitalized with P-EP to tertiary hospitals in high-income nations, displayed outstanding discriminatory performance, reaching an area under the receiver operating characteristic curve (AUROC) of 0.88 (95% confidence interval [CI]: 0.84-0.92) [11]. In present study the age group under 20 years, none of the women experienced AMO. For the age group of 20-25 years, 4 women (28.6%) experienced AO, while 16 women (34.8%) did not. The 25-30 years age group had the highest number of AO, with 7 women (50.0%) affected, compared to 19 women (41.3%) without AO. In the age group over 30 years, 3 women (21.4%) experienced AO, whereas 8 women (17.4%) did not. The p-value for the distribution across these age groups was 0.7244, indicating no statistically significant difference in AMO based on age. Our study also found that among the primigravida group, 6 women (42.9%) experienced AMO, while 21 women (45.7%) did not. In the group of women with two or more pregnancies, 8 women (57.1%) had AO, whereas 25 women (54.3%) did not. The p-value is 0.854, indicating no statistically significant difference in AMO between primigravida and multigravida women. This suggests that gravidity may not be a significant factor in predicting AMO in this study population. In present study among those with AO, 50.0% reported swelling, compared to 45.7% without AO (p = 0.7752). Right upper quadrant or epigastric pain was reported by 35.7% of women with AO, significantly higher than the 10.9% without (p = 0.0192). Chest pain or dyspnea was reported by 28.6% of women with AO compared to 10.9% without (p = 0.0401). Headaches were reported by 21.4% of those with AO and 21.7% without (p = 0.9803). Nausea and vomiting were noted in 14.3% of AO cases and 21.7% of non-adverse cases (p = 0.5416). Visual disturbances were reported by 14.3% of women with AO and 8.7% without (p = 0.5416). Lastly, 7.1% of women with adverse outcomes had no symptoms, compared to 19.6% of women without AO (p = 0.276). Study also found that, NICU admission was the most common adverse fetal outcome, affecting 14 cases (53.8%), followed by meconium-stained liquor (MSL) in 11 cases (42.3%). Ten cases (38.5%) experienced an APGAR score less than 4 at 5 minutes, indicative of compromised neonatal health immediately after birth. Fetal growth restriction was noted in 9 cases (34.6%), while 7 cases (26.9%) involved premature birth. There was one reported case each of stillbirth (3.8%) and neonatal death (3.8%). These findings underscore the significant impact of maternal health conditions on FO, with a substantial proportion of infants requiring intensive care and exhibiting signs of distress shortly after birth. In the study by Guida *et al.* the median age of the 208 women with P-EP /superimposed P-EP was 29 years and the majority of births were premature (<37 weeks) [12]. Sivakumar *et al.* conducted a study with 61 patients (48.8%) presented with swelling, making it the most common symptom. AMO were most frequently associated with chest pain or dyspnea, with 13 out of 24 women (54.16%) experiencing dyspnea also having negative outcomes. This was followed by headaches (34.2%) and blurred vision (22.22%) [13]. Martin *et al.* identified nausea, vomiting and epigastric pain as predictors of increased maternal morbidity [14]. Similarly, Cavkaytar *et al.* observed that in patients with HELLP syndrome, symptoms such as headaches, visual disturbances, epigastric pain and vomiting were more predictive of adverse maternal outcomes than laboratory values [15]. Additionally, Yen *et al.* found that P-EP symptoms alone do not reliably predict adverse maternal outcomes, advising caution in making clinical decisions based solely on these symptoms [16]. In a study by Ahmad *et al.* involving 377 neonates, several critical outcomes were observed. Of the neonates studied, 167 (44.30%) were born prematurely and 203 (53.85%) experienced fetal growth restriction. Additionally, 64 neonates (16.97%) had an Apgar score of less than 4 at birth, indicating severe distress and 67 neonates (17.77%) were born with meconium-stained amniotic fluid, suggesting potential fetal distress. A significant proportion, 90 neonates (25.50%), required admission to the NICU for further care. The study also reported intrauterine deaths in 24 neonates (6.37%) and neonatal deaths in 6 neonates (1.59%). These findings highlight the substantial challenges in neonatal health and emphasize the importance of targeted interventions to improve outcomes for high-risk pregnancies [17]. Guida *et al.* study reports several maternal and neonatal outcomes in high- risk patients. Eclampsia occurred in 8 patients (3.8%) and HELLP syndrome was observed in 14 patients (6.7%). Placental abruption affected 5 patients (2.4%), while there was 1 maternal death (0.5%). Neonatal outcomes included 7 cases (3.4%) where the 5th-minute Apgar score was below 7. NICU admissions were necessary for 92 new borns (44.2%), with an average total time spent in NICU being 15.4 days (±30.5 days). Additionally, there were 12 perinatal deaths (5.8%). This data underscores the significant maternal and neonatal complications associated with high-risk pregnancies, highlighting the importance of close monitoring and management to improve outcomes [12]. There were a total of 101 women who suffered maternal complications, 39.5% of the time, whereas 120 women (46.9% of the time) experienced fetal complications. On top of that, 159 women, or 62.1% of the total, had both sorts of complications. When it comes to predicting unfavorable maternal outcomes, the model exhibited a sensitivity of sixty percent and a specificity of ninety-seven percent, with a cut-off value of five percent or above. On the other hand, when it came to predicting combined fetomaternal complications, the sensitivity and specificity were 44% and 96%, respectively, when the cut-off value was 4.9%. Because of this, the authors decided that the full PIERS model does a good job of predicting bad outcomes for both the mother and the baby in women who have been diagnosed with pre-eclampsia [18]. It was found that the ratio of sFlt-1 to PlGF (377.0 cut-off) was significantly linked to the occurrence of complications for mothers (Spearman's rho, 0.728; p < 0.001). This ratio not only properly predicted these complications, but it also had a sensitivity of 75.0% and a specificity of 92.3%. The sFlt-1/PlGF ratio had superior predictive performance compared to PIERS, as shown by its area under the curve (AUC) value of 0.853 (95% confidence interval [CI]: 0.733-0.972; p < 0.001). The sFlt-1/PlGF ratio was the sole predictor variable in the final logistic regression model for predicting adverse maternal outcomes (odds outcome, 1.006 [95% CI 1.002-1.010]; p = 0.005). This was the only variable involved in the prediction of adverse maternal outcomes. When doctors do clinical exams and look at the sFlt-1/PlGF ratio, they might be able to better guess when bad things might happen to the mother [19]. The PREP models can tell you the chance of bad things happening to the mother, like giving birth early, within 48 hours (PREP-S) and by the time she leaves the hospital (PREP-L), especially for women who are starting to show signs of pre-eclampsia early in their pregnancy using the current system of care. Their potential role involves the triage of high-risk mothers who may require transfer to tertiary units for intensive maternal and neonatal care [20].

## Conclusion:

The PIERS score is both cost-effective and easy to use, offering reliable predictions of adverse outcomes. This makes it an excellent tool for healthcare workers at primary and secondary centers to identify pre-eclampsia patients at high risk for complications. Implementing this approach could significantly reduce maternal and perinatal morbidity and mortality associated with pre-eclampsia, especially in low- resource healthcare settings.

## Figures and Tables

**Table 1:** Maternal age distribution

**Maternal age in years**	**Adverse Maternal outcome Present**		**No Adverse Maternal outcome**		**P value**
	**Cases**	**Percentage**	**Cases**	**Percentage**	
<20 yrs	0	0.00%	3	6.50%	0.7244
20-25 yrs	4	28.60%	16	34.80%	
25-30 yrs	7	50.00%	19	41.30%	
> 30 yrs	3	21.40%	8	17.40%	
Total	14	100.00%	46	100.00%	

**Table 2:** Parity distribution

**Parity**	**Adverse Maternal outcome Present**		**No Adverse Maternal outcome**		**P value**
	**Cases**	**Percentage**	**Cases**	**Percentage**	
Primigravida	6	42.90%	21	45.70%	0.854
Gravida 2 or more	8	57.10%	25	54.30%	
Total	14	100.00%	46	100.00%	

**Table 3:** Symptoms distribution

**Symptoms**	**Adverse Maternal outcome Present (n=14)**		**No Adverse Maternal outcome (n=46)**		**P value**
	**Cases**	**Percentage**	**Cases**	**Percentage**	
Pedal edema	7	50.00%	21	45.70%	0.7752
Right upper quadrant or epigastric pain	5	35.70%	5	10.90%	0.0192
Chest pain or dyspnea	4	28.60%	5	10.90%	0.0401
Headache	3	21.40%	10	21.70%	0.9803
Nausea Vomiting	2	14.30%	10	21.70%	0.5416
Visual disturbances	2	14.30%	4	8.70%	0.5416
No symptoms	1	7.10%	9	19.60%	0.276

**Table 4:** Biochemical marker distribution

**Biochemical markers**	**Adverse Maternal outcome Present (n=14)**		**No Adverse Maternal outcome (n=46)**		**P value**
	**Cases**	**Percentage**	**Cases**	**Percentage**	
AST (> 40 U/l)	10	71.40%	15	32.60%	0.0098
Serum creatinine (> 1.1 mg/dl)	8	57.10%	8	17.40%	0.0032
Dipstick proteinuria (> 1)	7	50.00%	10	21.70%	0.0399
Platelet count (< 1.5 lacs)	6	42.90%	8	17.40%	0.0485

**Table 5:** GA at presentation

**Gestational age (GA) in weeks at presentation/ diagnosis**	**Adverse Maternal outcome Present**		**No Adverse Maternal outcome**		**P value**
	**Cases**	**Percentage**	**Cases**	**Percentage**	
< 34	2	14.30%	4	8.70%	0.0456
34-36	6	42.90%	13	28.30%	
37-39	3	21.40%	27	58.70%	
> 40	3	21.40%	2	4.30%	
Total	14	100.00%	46	100.00%	

**Table 6:** GA (time of delivery)

**Gestational age in weeks at the time of delivery**	**Adverse Maternal outcome Present**		**No Adverse Maternal outcome**		**P value**
	**Cases**	**Percentage**	**Cases**	**Percentage**	
< 37 week	2	14.30%	8	17.40%	0.9337
37-39	9	64.30%	36	78.30%	
> 40	3	21.40%	2	4.30%	
Total	14	100.00%	46	100.00%	

**Table 7:** Compare BP & AMO

**Blood Pressure**	**Adverse Maternal outcome Present (n=14)**	**No Adverse Maternal outcome (n=46)**	**P value**
	**Mean ± SD**	**Mean ± SD**	
Mean systolic blood pressure (MSBP) (at the time of admission) mmHg	170.2 ± 16.5	158.2 ± 17.2	0.0246
Mean diastolic blood pressure(MDBP) (at the time of admission) mmHg	104.9 ± 9.3	98.6 ± 8.9	0.0253
Mean arterial blood pressure (MABP) (mmHg)	127.6 ± 11.7	118.1 ± 10.8	0.0064

**Table 8:** Compare Mg_2_SO_4_ & AMO

**Patients required Mg_2_SO_4_**	**Adverse Maternal outcome Present**		**No Adverse Maternal outcome**		**P value**
	**Cases**	**Percentage**	**Cases**	**Percentage**	
Yes	8	57.10%	7	15.20%	0.002
No	6	42.90%	39	84.80%	
Total	14	100.00%	46	100.00%	

**Table 9:** Symptom & Adverse perinatal outcome (APO)

**Symptoms**	**Adverse perinatal outcome Present (n=26)**		**No Adverse perinatal outcome (n=34)**		**P value**
	**Cases**	**Percentage**	**Cases**	**Percentage**	
Pedal edema	10	38.50%	18	52.90%	0.2663
Headache	9	34.60%	4	11.80%	0.0332
Right upper quadrant or epigastric pain	7	26.90%	1	2.90%	0.0522
Chest pain or dyspnea	7	26.90%	2	5.90%	0.0237
Nausea Vomiting	6	23.10%	6	17.60%	0.6023
Visual disturbances	5	19.20%	1	2.90%	0.0371
No symptoms	3	11.50%	7	20.60%	0.3513

**Table 10:** Compare biochemical marker & APO

**Biochemical markers**	**Adverse perinatal outcome Present (n=26)**		**No Adverse perinatal outcome (n=34)**		**P value**
	**Cases**	**Percentage**	**Cases**	**Percentage**	**Percentage**
Platelet count (< 1.5 lacs)	11	42.30%	5	14.70%	0.0165
AST (> 40 U/l)	13	50.00%	12	35.30%	0.1088
Serum creatinine (> 1.1 mg/dl)	12	46.20%	4	11.80%	0.0028
Dipstick proteinuria (> 1)	10	38.50%	7	20.60%	0.1281

**Table 11:** Compare MOD & APO

**Mode of delivery**	**Adverse perinatal outcome Present (n=26)**		**No Adverse perinatal outcome (n=34)**		**P value**
	**Cases**	**Percentage**	**Cases**	**Percentage**	
Vaginal spontaneous	5	19.20%	21	61.80%	0.0079
Vaginal induced	13	50.00%	10	29.40%	
Caesarean section	7	26.90%	4	11.80%	

**Table 12:** Compare piers & AMO

**Parameters of full PIERS**			**Adverse maternal outcomes**				**P value**
		**Total Cases (n = 60)**	**Present (n=14)**		**Absent (n=46)**		
			**Cases**	**%**	**Cases**	**%**	
	< 34	6	2	14.30%	4	8.70%	0.0456
	34-36	19	6	42.90%	13	28.30%	
**Gestational age (in weeks)**	37-39	30	3	21.40%	27	58.70%	
	> 40	5	3	21.40%	2	4.30%	
	Present	9	4	28.60%	5	10.90%	0.0401
**Chest pain**	Absent	51	10	71.40%	41	89.10%	
	< 94.9	15	9	64.30%	6	13.00%	< 0.001
**SpO2 (in %)**	> 95	45	5	35.70%	40	87.00%	
	< 150,000	16	6	42.90%	8	17.40%	0.0485
**Platelet count/cumm**	> 150,000	44	8	57.10%	38	82.60%	
	< 1.1	16	8	57.10%	8	17.40%	0.0032
**Serum creatinine (mg/dL)**	> 1.1	44	6	42.90%	38	82.60%	
	< 40	25	10	71.40%	15	32.60%	0.0098
**Serum AST (IU/L)**	> 40	35	4	28.60%	31	67.40%	

**Table 13:** PIERS & AO

**PIERS Score**	**Number of women**	**Number of women with adverse outcome**	**% of women with adverse outcome**
< 30	53	9	16.98%
> 30	7	5	71.42%
Total	60	14	23.30%

**Table 14:** Validity

**Validity Statistics**	**Value**	**95% Confidence Interval**
Sensitivity	35.71%	12.76% to 64.86%
Specificity	95.65%	85.16% to 99.47%
Positive Likelihood Ratio	8.21	1.78 to 37.81
Negative Likelihood Ratio	0.67	0.45 to 1.00

**Table 15:** AMO

**Adverse maternal outcome**	**Cases**	**Percentage**
Thrombocytopenia	6	42.90%
Eclampsia	5	35.70%
PPH	5	35.70%
Abruption placenta	4	28.60%
HELLP syndrome	4	28.60%
ICU admission	4	28.60%
Ionotropic Support	4	28.60%
Acute Renal Failure	3	21.40%
Required blood / blood product	2	14.30%
Pulmonary Oedema	1	7.10%
Cerebral Vascular Accident	1	7.10%
Death	0	0.00%

**Table 16:** AFO

**Adverse fetal outcome (AFO)**	**Cases (n=26)**	**Percentage**
NICU Admission	14	53.80%
MSL	11	42.30%
APGAR < 4	10	38.50%
Foetal growth restriction	9	34.60%
Prematurity	7	26.90%
Still birth	1	3.80%
Neonatal Death	1	3.80%
